# Manufacture of fresh cheese using east African *Calotropis procera* leaves extract crude enzyme as milk coagulant

**DOI:** 10.1002/fsn3.1765

**Published:** 2020-08-13

**Authors:** Bethelhem Abebe, Shimelis Emire

**Affiliations:** ^1^ Addis Ababa Institute of Technology School of Chemical and Bio‐Engineering Food Process Engineering Program Addis Ababa University Addis Ababa Ethiopia

**Keywords:** acute toxicity, *Calotropis procera*, milk coagulation time, soft cheese, total milk‐clotting activity

## Abstract

The present work was aimed to develop fresh cheese using the crude extract from *calotropis procera* leaves and thereby performed evaluating milk‐clotting activity and acute toxicity of crude extract. The extraction of coagulant was performed at 30, 45, and 60℃ using 2, 6, and 10 g of dried *calotropis procera* leaves, respectively. The highest yield of crude enzyme extract was acquired with 2 g and 30℃, and results were varied significantly (*p* < .05). The highest cheese yield and fastest clotting time were 17.89 kg cheese/100kg milk and 14:50 min acquired with 10 g *C. procera* powder at 60℃ extraction temperature, respectively. *E. coli*, total bacterial count, yeast, and mold microbial load detection were observed below the limit established by codex alimentarius. This study recommends that use of *C. procera* crude enzyme leaves extract can be used as alternate milk coagulant for production of fresh cheeses. Soft cheeses manufactured with *calotropin* enzyme as milk coagulant revealed no adverse effects of toxicity tested on albino rates to the highest dose.

## Graphical abstract

This research study confirms that the soft cheese produced using calotropin enzyme as milk coagulant revealed no adverse effects of toxicity. Thus, the practical aspects can contribute to food and nutrition security in Africa and Asia. The East African countries can open their senses of opportunity to manufacture various commercial enzymes for dairy and other food manufacturing industry from plant and microbial sources.

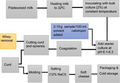

## 1 INTRODUCTION

Cheese is a solid milk product obtained from milk of cow, goat, sheep, and other mammals obtained by curdling milk using rennet addition, acidification, or a combination of rennet and acidification methods (Ogunleke, 2015). Cheese manufacture is essentially a dehydration process in which the fat and casein in milk are concentrated between 6‐fold and 12‐fold, depending on the variety (Fox, McSweeney, Cogan, & Guinee, 2017).

The coagulation of milk by enzymatic methods is a basic step in the manufacture of most cheeses. The addition of rennet or coagulating agents has been greatly used in the coagulation of milk for the production of cheese. Chymosin is the principal milk‐clotting enzymes present in the natural calf rennet and still the most extensively used milk‐clotting enzyme yet in the cheesemaking process. It is active enzyme in rennet, extracted and purified from the stomach of unweaned calves involving many steps which make the enzyme expensive (Naz, Masud, & Nawaz, 2009). Nowadays, most commercial rennet used in the cheese industry comes from recombinant sources or from microbial origin, and only 20%–30% comes from its natural source (Jacob, Jaros, & Rohm, 2011).

Worldwide increase in cheese production, coupled with reduced supply and increasing prices of calf rennet, has led to search for alternative milk‐clotting enzymes, as appropriate rennet substitutes (Anusha, Singh, & Bindhu, 2014 and Shah, Mir, & Paray, 2014). Apart from this, some religious factors (Islam and Judaism) and others related to vegetarianism of some consumers have greatly limited calf rennet use (Shah et al., 2014; Amal *et al*., 2017). In generally, the decline in the natural rennet supply (stomach of calves) and the stable increase in world cheese processing in recent years have led to an augmented demand for new rennet substitutes, motivating a search for new sources of proteases with rennet‐like properties from plant sources. Numerous attempts have been made to replace expensive animal rennet with low‐priced plant substitutes in order to deal with its shortage, but unfortunately most of them are considered unsuitable either due to their excessive proteolytic nature or extreme bitterness in final cheese products (Naz et al., 2009).

Several milk‐clotting enzymes of microbial origin have been commercialized and used in cheese processing. Nowadays, plant rennets have become a subject of growing interest in cheese industry, due to their easy availability and simple purification processes (Milica, Lidija, & Marija, 2013). Furthermore, the use of plant proteases in cheese manufacturing promotes the greater acceptability by the vegetarians and may improve their nutritional intake (Duarte et al., 2009). For several years, plant extracts have been widely used in the preparation of various types of cheeses which are mainly produced in Mediterranean countries, Southern Europe, and West Africa (Roseiro, Barbosa, Mames, & Wilbey, 2003).

Crude extracts from fruits and plants have been used for long time as milk coagulating agents. These include extracts from papaya (papain), pineapple (bromelain), castor oil seeds (ricin), the latex of the fig tree, and the plant *calotropis procera* which grows abundantly in many parts of Africa. The proteases from plant sources offer a high potential as processing aids in production of cheese with characteristic aroma and texture to cheeses. The common plant proteases papain, ficin, and bromelain have a low milk‐clotting activity/proteolytic activity (MCA/PA) ratio and have often been mentioned as the principal obstacle to their utilization in cheesemaking (Feijoo‐Siota, Villa, Feijoo‐Siota, & Villa, 2011). A broad proteolytic specificity and the nonspecific hydrolysis of caseins affect texture, flavor, and yield of cheese (Jacob et al., 2011). However, some proteases that are highly specific for milk proteins are attractive as milk‐clotting and/or ripening agents in cheese production.

The high prices of calf rennet, refusal to accept cheese made from animal rennet in general and porcine rennet by vegetarians and Muslims, respectively, necessitated the need to substitute animal rennet with easily available, relatively cheap, and acceptable source of rennet for cheese preparation. For these reasons, the plant *Calotropis procera* has been given much attention for alternative vegetable rennet since it has the potential to completely substitute animal and microbial rennet used for commercial cheese production. Extracts from the succulent leaves and stems of *C. procera* have been reported to contain rennet enzymes called *calotropin* that coagulates milk (Chikpah, Teye, Teye, & Mawuli, 2014).

*Calotropis procera* is a wild African bush potential plant, which is rich in bioactive substances (phytochemicals). The plant has high activity of antimicrobial, antioxidant, antifungal, antidiabetics, and anticancer, and it is one of traditional medicinal plant in Ethiopia. The *Calotropis procera* plant grows in dry deciduous bush lands, plain soil at roadsides, and abandoned residences. The plant grows widely on the altitude around 600–2,300 m in Afar plains, Shoa, Wello, Gojam, Tigray, Harerghe, Arsi, Sidamo, Gamo Gofa, and Iliababra parts of Ethiopia. C. procera is a traditional herbal plant and named in different parts of Ethiopia such as Qimbo, Tobiaw, Ghinda (Amharic), Falfala adal (Oromipha), Akalo, Dinda (Tigregna), Abuwo (Agnuak), and Boha (Somalic), and in English, it is named as Apple of Sodom (Dead Sea fruit) (Bekele, 2007).

*Calotropis procera* leaves traditionally use as antimicrobial activity in flat bread handling in pastoral and agro‐pastoral areas of Ethiopia. The *calotropine* is a protease cysteine coagulant agent which plays a great role in milk‐clotting activity it presents in the *C. procera* plant parts (Benyahia Krid et al., 2016). Dairy food spoilage is caused by the growth of microorganisms, primarily bacteria and fungi, that convert nutrients into energy which they use for their own growth. Depletion of the nutrient content of dairy food and the secretion of by‐products from this biochemical process are two things which contribute to the spoilage of food rendering it inedible. The most common microbial defects of cheese are the development of early and late gas, but these problems were overcoming by better hygiene in milk production and better quality control in cheese plants (Hayaloglu, 2015). Knowing the microbial quality profile in soft cheese processing is very vital prior to sensory quality analysis in this research. The more control you have over the microbes, the more consistent your cheese will taste.

According to Kamel, Morsy, Zinecker, Imhoff, and Schneider (2010), *calotropis procera* has biologically active substances such as flavonoids, cardioactive glycosides, triterpenoids, alkaloids, resins, anthocyanins, tannins, saponins, and proteolytic enzymes. *Calotropis procera* is a plant widely distributed in tropical and subtropical regions of Africa and Asia with a long history of use in traditional medicine. The *Calotropis procera* has been shown to possess some coagulating properties and has been used as a coagulant in soft cheese (*ayib*) making by the Raya Agro‐Pastoralists, particularly in *Mehoni*, *Chercher*, *Megalle, Harra,* and nearby the Kobo and Zoble mountain of northeastern part of Ethiopia.

While the potential exists for using plant proteases more extensively in dairy food processing and other biotechnological processes, information regarding their development as processing aids, particularly their utility in cheesemaking, is scarce. Accordingly, the aim of the present research was to evaluate the milk‐clotting activities of *C*. *procera* crude enzyme leaves extract in terms of temperature dependence and amount of dried *calotropis procera* leaves and their performance on coagulating time, cheese yield, and quality characteristics of soft cheese. Besides, toxicity of *calotropis procera* extract crude enzyme on albino rats was also evaluated.

## 2 MATERIAL AND METHODS

### 2.1 Collection of *Calotropis procera* fresh leaves

The succulent leaves of *Calotropis procera* plant were collected from Nazareth (90 km southeast of Addis Ababa); it is located 8°33′0″ N Latitude, 39°16′0″ E Longitude at an elevation of 1,627 m with an average annual rainfall of 808 mm. The collected *C. procera* leaves were, identified by Botany Department, cleaned very well and dried by setting the temperature at 45 ± 1°C according to the method described by Netoa et al. (2013). Then after, dried leaves of *Calotropis procera* ground into powder by using an electric blender and kept the powder under airtight container until next use.

### 2.2 Coagulant extraction

The *C. procera* leaves powder was weighed in portions of 2, 6, and 10 grams using the method described by Chikpah et al. (2014), by increasing the sample dose based on the sideline laboratory toxicity test. The extraction was assessed by the method described by García et al. (2012) and Shobowale, Ogbulie, Itoandon, Oresegun, and Olatope (2013). The extraction temperature was modified from constant temperature to 30, 46, and 60°C for each sample dose, to analyze crude extract on clotting activity and cheese manufacturing process.

### 2.3 Processing of soft cheese

**Figure 1:**
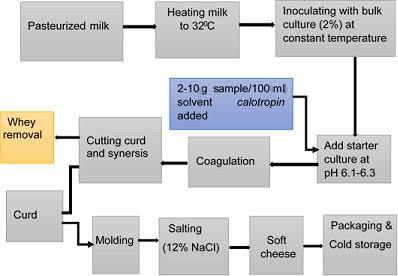
Flow diagram of soft cheese production according to Fox et al. (2017)

The development of soft *C. procera* cheese was prepared following the general soft cheese production procedure described by Fox, Guinee, Cogan, and Mcsweeney (2017) (Figure 1). Each cheese sample was made from 2,000 ml pasteurized cow milk with protein and fat content of 3.5% and 2.7%, respectively. Cheese vat temperature was set at 32°C, and the probiotic starter culture was added at a rate of 1.5 ml/100 ml at set temperature. Then, it was incubated for about 4–5 hr. When the pH reached to 6.1–6.3, the prepared crude *calotropin* was added to the milk and stirred to enhance distribution of enzyme throughout the cheese vat and kept until curd formation was apparent. After checking curd strength by using spatula, the curd mass was cut using stainless steel cheese knife at pH 4.8 to remove the whey and drained further using cheese cloth and mesh sieve. After whey was drained, the curd was molded in shapes and finally molded soft cheese was dipped in a brine solution of 12% NaCl for about 12–15 hr, which was performed to impart a good test for the cheese and as a preservative. Samples coded (C1, C4, C7), (C2, C5, C8), and (C3, C6, C9) are cheese samples made at concentration (dose) of 2, 6, and 10 g of *C. procera* powder extract enzyme, respectively. C1 to C9 are cheese produced by *C. procera* crude extract enzyme.

Finally, different analysis for developed soft cheese samples was performed comprising yield, moisture, texture, color, compositional, microbial, and sensory quality attributes using standard procedure. The following flowchart was generally employed to produce soft cheese production.

### 2.4 Acute toxicity evaluation of crude enzyme extract and soft cheese

Acute toxicity of *C. procera* extract and cheese developed by crud extract were performed on albino mice as method described by OECD guideline 420 (OECD, 2001) and according to Netoa et al. (2013). Experimental animals used in this study were thirty‐five healthy female mice (weighing 20–25 g) and were obtained from animal house of Ethiopian Public Health Institute. They were kept under standard conditions (at a temperature of 21 ± 2℃, with 12 hr’ light/12 hr’ dark cycle) and provided with free access to standard pellet laboratory diets and drink tap water unlimitedly. Before the experiment, they were grouped randomly into 7 groups (*n* = 5/group) and then kept in their cages for 5 days to allow acclimatization to the laboratory conditions.

After acclimatization, all groups were fasted. Doses and the vehicle were calculated based on their body weight. Conversion of human to animal dose calculation was performed according to Shin, Seol, and Son (2010) and Nair (2016). The *C. procera* extract was then administered orally, using oral gavage, at a single dose of 26.84, 46.97, 67.1, 93.94, and 134.2 mg/kg (with conversion factor) for albino mice in the test group of 1, 2, 3, 4, and 5, respectively. The control group (7) received distilled water, and the other group (6) were fed with cheese made using *C. procera* extract instead of pellet (standard laboratory diets) for 14 continuous days. The observations were performed during the first four hours after the oral treatments to determine LD_50_ and to assess the effect on behavior. After that, clinical observations were made once every 24 hr for the next 14 days for mortality, behavior (piloerection, tremors, sedation, loss of corneal reflex, motor activity), body weight, and gross physical changes. Finally, on the 15th day, their final weights were measured, and then, they were sacrificed to examine gross pathological changes in their organs.

### 2.5 Measurement of milk‐clotting activity

The method of Berridge (1952) was used to measure the clotting activity in rennet unit (RU). The milk‐clotting activity of *C.procera* crud enzyme extract was measured for nine different extracts (E1‐E9) at various temperatures (32, 50, and 60℃). The standard substrate was prepared by reconstituting skim milk powder at 10% (w/v) solution of 0.01 M CaCl_2_ and warmed at 32℃. The prepared extracts were added at a proportion of 1 ml/10 ml of standard substrate, mixed manually, and incubated in a water bath at 32°C. After thoroughly mixing three times, the “zero” clotting time started. The milk‐clotting activity of each extract was measured, with the assumption that all the soluble proteins from the extract were enzymes which coagulate milk at 32, 50 and 60℃. The clotting activity equation as reported by Berridge (1952) in rennet units (RU) was used.

(1) $$RU=\frac{10*V}{TC*Q}$$

where RU‐rennet unit; V–volume of standard substrate (ml); Tc–clotting time (second); Q–volume of plant crude extract (mL).

### 2.6 Compositional analysis of milk and fresh cheese

#### 2.6.1 Compositional analysis of milk

The crude fat, protein, and lactose of the pasteurized milk were determined by using milk scan instrument, while the ash content was determined according to AOAC (2000) official method no.923.03. The pH of the pasteurized milk was determined based on the standard methods of AOAC (2000).

#### 2.6.2 Proximate and physicochemical analysis of soft cheese

##### Protein

Protein content of cheese samples was determined according to the method described by Ogunlade, Oyetayo, and Ojokoh (2017).

(2) $$\text{Nitrogen}\;\left(\%\right)=\frac{\text{Vol}\text{.of}\;H_{2}{\text{SO}}_{4}\left(\text{ml}\right)\times 250\times 0.0014}{\text{Vol}\text{.of}\;\text{used}\;\text{for}\;\text{digestion}\times \text{vol}\text{.of}\;\text{digested}\;\text{sample}}\;\times 100$$

##### Fat

Fat content determination was carried out by Soxhlet extraction method based on AOAC (2000), Method no. 933.05.

##### Ash

Ash content determination was performed according to AOAC (2000), Method no. 923.03.

(3) $$\text{Ash}\;\left(\%\right)=\frac{\text{wt}\;\text{after}\;\text{ashing}‐\text{tare}\;\text{wt}\;\text{of}\;\text{crucible}}{\text{original}\;\text{sample}\;\text{wt}*\;\text{dry}\;\text{matter}\;\text{coefficient}}*100$$

where dry matter coefficient = % solids/100.

#### 2.6.3 Mineral composition

The mineral composition of developed soft cheese was analyzed according to the method described by Hui (2006) using the atomic absorption spectrophotometry.

### 2.7 Color and Titratable acidity

#### 2.7.1 Color analyses

The color in cheese was measured using color flex spectrocolorimeter after being standardized using a Hunter Lab color stabilizer. The unripened cheese samples were placed beneath the optical sensor at the tip of the instrument and allowed for postprocessing. The parameters recorded were L*, a*, and b* coordinates of the CIE scale was performed according to Netoa et al. (2013).

#### 2.7.2 Titratable acidity

For determination of titratable acidity, about 10 g cheese was weighed and crushed with 105 ml water. This solution wasLLac filtered, and 25 ml of filtered solution was used for titration. Three drops of phenolphthalein were added and titrated with 0.1 N NaOH until the first permanent pink color was appeared it was performed according to Endale, Eshetu, and Zelalem Yilema (2016).

(4) $$\text{Lactic}\;\text{acid}\;\left(\%\right)=\left[\frac{\text{ml}\frac{N}{10}\text{NaOH}*0.009}{\text{ml}\;\text{of}\;\text{sample}\;\text{used}}\right]100$$

### 2.8 Cheese yield, clotting time and texture profile

Cheese yield, clotting time, and texture profile analyses were performed for ten cheese samples produced using commercial rennet and *C. procera* crude extract enzyme.

#### 2.8.1 Cheese yield

Cheese yield was calculated according to the method described by Akinloye and Adewumi (2016) and Paul et al. (2017). The actual cheese yield was calculated based on the equation given below. The yield and actual cheese yield were expressed as weight of cheese curd per liter of milk used (g/L) and kg cheese/100 kg milk.

(5) $$\text{Ya}=\frac{\text{grams}\;\text{of}\;\text{cheese}\;\text{produced}}{\text{grams}\;\text{of}\;\text{milk}\;\text{used}+\text{starter}\;\text{culture}}*100\%$$

where Ya = actual cheese yield.

Ya expressed in kg cheese/100 kg milk.

#### 2.8.2 Clotting time for cheese production

The time taken for the pasteurized cow milk to make a first clot formation was observed carefully and recorded. The average time taken for coagulation was taken as a clotting time for each samples according to the method described by Chikpah and Teye (2015). Milk‐clotting activity of crude enzyme extract was assessed at different temperatures. The clotting time was determined by dipping a clean spatula or glass slide into the milk. When coagulation has occurred, flecks of curd were appeared in the milk film on the slide.

#### 2.8.3 Texture profile

Texture profile analysis (TPA) of soft cheeses was performed according to the method described by García et al. (2012). Texture Analyzer (LLOYD Instruments, TA plus Ametek, UK 2007) was used to determine the hardness of soft cheeses. The sample was wrapped and equilibrated at 20℃ for 30 min before testing. The cheese was placed in the center of the plate, and then, cutting jig probe was applied at constant crosshead speed of 21 mm/s.

### 2.9 Microbiological quality of soft cheese

Microbiological analyses of fresh cheese samples were conducted based on Adetunji and Babalobi (2011). It was performed for four cheese samples, where sample 1, 2, and 3 are produced at minimum, medium, and maximum concentrations of coagulant and extraction temperature whereas sample 4 was a control cheese produced by commercial rennet. Each four samples of cheeses were analyzed in triplicate at initial day of production. *E. coli*, yeast, mold, and total aerobic plate counts (TPC) were determined using the method NMKL No. 125 (2005) and NMKL No. 98 (2005) and NMKL No. 86 (2006), correspondingly. The number of colonies on the tested sample was described as colony forming unit cfu/g.

(6) $$N=\frac{\sum C}{\left[\left(1\times n_{1}\right)+\left(0.1\times n_{2}\right)\times \left(d\right)\right]}$$

where *N* = the average colonies per ml or g of product; C = sum of all colonies on all plates counted; *n*_1_ = number of plates in the first dilution counted; *n*_2_ = number of plates in the second dilution counted; d = dilution from which first count was obtained.

### 2.10 Sensory attribute analyses of fresh cheese

Three soft cheese samples of equal weight were designed to sensory evaluation using 35 unexperienced panelists based on a five‐point hedonic scale from 1 to 5 (1‐ "disliked" to 5 –"excellent") according to Adetunji and Babalobi (2011). The sensory quality attributes considered taste, flavor, color, texture, and overall acceptability of products.

### 2.11 Experimental design and statistical data analysis

This design of the research experiment used was completely randomized design. ANOVA of 95% confidence interval and Design Expert software version 7.0 were used. In addition, the data obtained were statistically analyzed by using SPSS software version 20.

## 3 RESULTS AND DISCUSSION

### 3.1 Acute toxicity of crude extract and manufactured fresh unripen cheese

**Figure 2:**
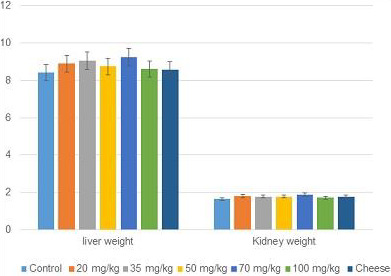
Comparison of weight of treated mice organ with different doses for acute toxicity test

In toxicity study, the extract of leaves up to the highest dose of 134.2 mg/kg did not show any significant changes on behavior (such as alertness, aggressiveness, and irritability), gross physical appearance (condition of fur, general cleanliness) compared with the control (Figure 2). The gross pathological examination on the organ of treated albino mice showed no significant changes in color, size, shape, and texture compared with the control group based on laboratory observations (Figure 2). The extract of *C. procera* and cheese developed by *C. procera* crude extract did not induce changes in the behavior of mice. No death was recorded during the 14 days’ test. Crude *C. procera* aqueous extract and the cheese LD50 could not therefore be estimated and are possibly higher than 134.2 mg/kg.

Treated albino mice organs (liver and kidney) were not significant (*p* > .05) statistically as compared with the control groups feed with distilled water as control (Table 1). There was a gradual increase in the body weight of both the treated and control mice. At the end of the experiment (after 14 days), the mean body weight gain for mice treated with different doses, control diet, and soft cheese was presented in Table 1.

**Table 1:** Effect of *C. procera* extract crude enzyme on albino mice body weight gain

Doses	Initial body weight (g)	Final body weight (g)	Mean weight growth (g)	Weight gain (%)
Control diet	22.54 ± 1.48^c^	28 ± 1.76^c^	5.46	24.22
20 mg/kg	22.68 ± 1.16^c^	29.44 ± 1.64^b^	6.76	29.80
35 mg/kg	24.08 ± 2.21^a^	30.46 ± 1.58^a^	6.38	26.49
50 mg/kg	23.1 ± 1.76^b^	28.68 ± 2.61^c^	5.58	24.15
70 mg/kg	23.2 ± 1.82^b^	29.72 ± 2.36^b^	6.52	28.10
100 mg/kg	23.58 ± 1.08^b^	30.44 ± 1.27^a^	6.86	29.09
Soft Cheese	22.08 ± 2.34^c^	25.7 ± 2.38^d^	3.62	16.39

Note

All values are means ± *SD*. Values with the same subscripts of same column are not significantly (*p* > .05) different, where control diet is distilled water.

### 3.2 Crude *C. procera* extract yield and pH

The yield of crude *C. procera* extract as coagulant ranged 85.04–57.74 ml at extraction temperature of 30°C and pH value of crude *C. procera* extract arrayed between 6.69 and 6.41 (Table 2). Results of *C. procera* extract pH value were moderately close to the finding reported by Akinloye and Adewumi (2016). According to the research findings reported by Akinloye and Adewumi (2016), the *Calotropis procera* plant coagulant yield of extract was 666 ml which is more value compared to the current findings (85.04 ml). The pH and yield higher values for *Calotropis procera* extract were 6.71 and 85.04 ml, respectively. In general, the crude *C. procera* extract yield can depend on the origin of the plant material. extraction temperature, environmental condition, moisture content, rain fall pattern, plant management, and dose applied.

**Table 2:** Yield of crude *C. procera* extract and pH value

Extraction temperature (℃)	Sample weight(g)	Yield of extract (ml)	pH
30	2	85.04^a^	6.69 ± 0.09^a^
	6	70.84^b^	6.65 ± 0.11^a^
	10	57.74^d^	6.56 ± 0.09^a^
45	2	81.44^a^	6.71 ± 0.09^a^
	6	67.44^c^	6.67 ± 0.16^a^
	10	64.24^c^	6.47 ± 0.04^b^
60	2	77.64^b^	6.46 ± 0.06^b^
	6	72.14^b^	6.41 ± 0.07^b^
	10	71.74^b^	6.42 ± 0.09^b^

Note

All values are in means ± *SD*. Values with the same subscripts of same column are not significantly (*p* < .05) different.

### 3.3 Milk‐clotting activity of crude enzyme extract

The milk‐clotting activity of crude *C. procera* extract is shown in Table 3. The results are ranged from 0.055 RU for extract one (E_1_) where milk‐clotting activity test performed at 32℃ to the highest 0.160 RU for extract nine (E_9_) which performed at 60°C.

**Table 3:** Milk‐clotting activity of crud *C. procera* extract expressed in rennet unit (RU)

Sample	RU (32℃)	RU (50℃)	RU (60℃)
E_1_	0.055 ± 0.001^c^	0.079 ± 0.003^d^	0.090 ± 0.001^e^
E_2_	0.056 ± 0.003^c^	0.082 ± 0.001^c^	0.101 ± 0.002^d^
E_3_	0.059 ± 0.001^b^	0.085 ± 0.002^b^	0.110 ± 0.002^c^
E_4_	0.056 ± 0.001^c^	0.081 ± 0.002^c^	0.095 ± 0.001^e^
E_5_	0.059 ± 0.003^b^	0.082 ± 0.001^c^	0.102 ± 0.002^d^
E_6_	0.060 ± 0.003^a^	0.086 ± 0.002^b^	0.115 ± 0.001^c^
E_7_	0.057 ± 0.001^c^	0.085 ± 0.001^b^	0.101 ± 0.001^d^
E_8_	0.063 ± 0.003^a^	0.092 ± 0.003^a^	0.149 ± 0.003^b^
E_9_	0.065 ± 0.002^a^	0.092 ± 0.003^a^	0.160 ± 0.004^a^

Note

All values are in means ± *SD*. Values with the similar subscripts of same column are significantly(*p* < .05) different, where E_1_, E_4,_ and E_7_ are crude extracts of 2g sample/100 ml solvent extracted at 30, 45, and 60°C, respectively;E_2_, E_5_ and E_8_ are crude extracts of 6g sample/100 ml solvent extracted at 30, 45, and 60°C, respectively; and E_3_, E_6,_ and E_9_ are crude extracts of 10 g sample/100 ml solvent extracted at 30, 45, and 60°C, respectively.

From the current study, optimum milk‐clotting activity endured 60 ℃ and it was in agreement with the findings of Ishak et al. (2006), Oseni and Ekperigin (2013), and Tajalsir et al. (2014) who have found the optimum temperature of plant coagulant milk‐clotting activity was close to 60–70℃ for Kesinai leaves, *C*. *procera,* and various plant coagulants, respectively. Highest milk‐clotting activity of current study was 0.160 RU which is comparable with clotting activity of Bovine rennet (0.179 RU) reported by Endale et al. (2016), and lowest clotting activity (0.055 RU) was moderately higher when it compared with the result of bovine rennet (0.023 RU). The current result of MCA was in agreement with that of Guiama et al. (2010) and AmiraBesbes, Attia, and Blecker (2017). The clotting activity from crude extract of *C. cardunculus* was 0.164 RU/ml. Accordingly, result of Ishak et al. (2006) *Kesinai* leaves clotting activity was 0.6, 0.65, and 0.7 MCA at 60, 65, ad 70℃, respectively. When concentration of extract and temperature increases, milk‐clotting activity increased for plant extracts.

### 3.4 Compositional analyses of milk and fresh cheese

#### 3.4.1 Compositional analysis of fresh cheeses

Nine different cheese samples compositional analysis such as moisture content, total solids, fat, protein, and ash (%) was analyzed and ranged between 56.34 and 65.38, 34.62 and 43.66, 11.08 and 16.86, 17.55 and 30.53, and 3.02 and 4.52%, respectively (Table 4). Sample coded with C3, C6, and C9 shows the highest fat and protein content, which is high dose treated with high temperature. Compositional analysis of the unripened cheese obtained for all samples in the current study was more or less in conformance with the findings of Uaboi‐Egbenni et al. (2010). Where the moisture content of 64%, 12.86% protein, 13.4% fat, and 0.60% ash was reported for a cheese made with the extract of Sodom apple (*Calotropis procera)* leaf extract and lactic acid.

**Table 4:** Compositional analysis of fresh cheese based on dry weight basis

Sample	Moisture (%)	Total solids (%)	Fat (%)	Protein (%)	Ash (%)
C_1_	56.34 ± 3.1^d^	43.66 ± 3.11^a^	13.04 ± 4.21^d^	19.37 ± 4.11^b^	3.02 ± 0.60^d^
C_2_	63.99 ± 2.5^a^	36.01 ± 2.93^c^	12.99 ± 2.32^d^	17.80 ± 3.34^c^	4.15 ± 1.00^b^
C_3_	59.48 ± 3.2^c^	40.52 ± 4.04^b^	16.86 ± 2.24^a^	18.49 ± 2.43^b^	3.51 ± 0.76^c^
C_4_	59.19 ± 4.1^c^	40.81 ± 2.16^b^	15.69 ± 4.23^b^	17.94 ± 2.07^c^	4.36 ± 0.87^a^
C_5_	65.38 ± 3.0^a^	34.62 ± 3.23^d^	14.00 ± 2.52^c^	18.14 ± 2.67^b^	4.52 ± 0.98^a^
C_6_	61.59 ± 2.1^b^	38.41 ± 2.54^c^	15.49 ± 4.33^b^	18.25 ± 3.87^b^	4.07 ± 0.67^b^
C_7_	56.54 ± 2.6^d^	43.46 ± 2.23^a^	11.08 ± 2.33^e^	17.55 ± 2.45^c^	4.33 ± 0.99^a^
C_8_	58.32 ± 3.9^c^	41.68 ± 2.64^b^	11.38 ± 4.21^e^	18.46 ± 3.22^b^	3.30 ± 0.76^c^
C_9_	62.67 ± 1.0^b^	37.33 ± 4.02^c^	14.53 ± 2.32^c^	30.53 ± 2.45^a^	3.53 ± 0.89^c^

Note

All values are means ± *SD*. Values with the same subscripts of the column are significantly(*p* < .05) different, where samples coded (C_1_,C_4_,C_7_), (C_2_,C_5_,C_8_), and (C_3_,C_6_,C_9_) are cheese samples made at concentration (dose) of 2, 6, and 10 g of c.procera crude extract enzym,; respectively. C_1_ to C_9_ are cheese produced by c.procera crude extract enzyme.

Other findings on wara soft cheese produced by *C. procera* extract were reported by Adetunji and Babalobi (2011) who stated result of wara protein, fat, and moisture was 12.56, 14.43, and 62.89%, respectively. According to the research findings reported by García et al. (2012), the protein content of unripened cheese made from the plant rennet extracts of *Cynara cardunculus* subspecies *Cardunculus* and *Cynara cardunculus* subspecies flavescens has less values compared to current research findings of cheese made from *C*. *procera*. Generally, *C. procera* coagulant significantly (*p* < .05) affects the compositional analyses of cheese, which in turn indicates that as the dose of *C. procera* increases, the content of protein and fat also increases too. Although ash content also increased in the manufactured soft cheese products which in turn indicate *C. procera* cheese might be rich source of minerals.

#### 3.4.2 Mineral composition

The processed soft cheese contains essential minerals such as calcium, iron, and zinc. From Table 5, it is evident that calcium content of the soft cheese ranged from 6.78 to 312.07 mg/100 g. Similarly, zinc and iron contents were ranged from 1.41 to 2.50 mg/100 g and 0.07 to 1.85 mg/100 g, respectively.

**Table 5:** Mineral concentration of soft cheese expressed in mg/100g

Extraction temperature	Samples	Ca (mg/100 g)	Fe (mg/100 g)	Zn (mg/100 g)
T_1_	C_1_	40.64	0.23	1.99
	C_2_	210.16	0.39	2.09
	C_3_	135.14	0.38	2.29
T_2_	_C4_	271.36	0.65	1.74
	C_5_	312.07	0.46	1.83
	C_6_	183.60	0.46	1.41
T_3_	C_7_	128.21	0.45	2.50
	C_8_	78.40	0.07	1.62
	C_9_	6.78	1.85	1.80
	C_10_	108.25	0.95	2.15

Note

All values are means ± *SD*; values with the same subscripts of the alike column are significantly (*p* < .05) different. Where T_1_, T_2_, and T_3_ are extraction temperature of crude extract enzyme at 30, 45, and 60°C, respectively; where C_10_ (cheese 10) represents control sample produced using commercial rennet and the rest cheese 1–9 (C_1_‐C_9_) were cheeses produced by c.procera crude extract; and where samples coded (C1, C4, C7), (C2, C5, C8), and (C3, C6, C9) are cheese samples made at concentration (dose) of 2, 6, and 10 g of c.procera crude extract enzyme, respectively.

Fresh cheese developed by *C. procera* had increased mineral content comparing to control cheese. This is due to the presence of minerals in the plant coagulant. Thus, the calcium is an important for osteoporosis and bone disease, as it is the major building block of human bone tissue. Recommended daily average intake of calcium is 1,000–1,300 mg/day stated by international osteoporosis foundation (IOF). Thus, the required amount of calcium can be obtained from fresh cheese of *C. procera* coagulant.

The calcium content obtained in this study was in agreement with the findings of Roseiro et al., 2003, in which calcium contents at two dairy plants were 13.11 and 12.48 g/kg. The finding of iron content was moderately higher compared with research findings of Malomo, Faduola, Adekoyeni, and Jimoh (2013) and which was 32.7 ppm. Although it is more likely close with the findings of Adetunji and Babalobi (2011). Moreover, control cheese sample mineral composition was less value compared with the soft cheese samples prepared using crude enzyme extract of *C. procera*.

#### 3.4.3 Color and titratable acidity of soft cheese

Titratable acidity and color indices (L*, a*, and b* values) and pH of the three cheese samples were presented in Table 6. Result of pH varied from 6.12 to 6.35, titratable acidity varied from 0.104% to 0.108%, and color indices L* (88.26–84.1), a* (0.78–2.33), and b* (25.68–18.98) as shown in the Table 6. All values were differed significantly (*p* < .05).

**Table 6:** pH, titratable acidity, and color of selected cheese samples

Samples	pH	Titratable acidity	L*	a*	b*
C_1_	6.35 ± 0.03^a^	0.106 ± 0.001^b^	88.26 ± 1.22^a^	0.78 ± 0.03^c^	25.68 ± 1.47^a^
C_2_	6.27 ± 0.05^b^	0.108 ± 0.001^c^	84.1 ± 0.00^c^	2.33 ± 0.02^a^	20.45 ± 2.9^b^
C_3_	6.12 ± 0.01^c^	0.110 ± 0.003^a^	86.92 ± 2.64^b^	0.81 ± 0.01^b^	18.98 ± 0.34^c^

All values are means ± *SD*. Values with the alike subscripts of the same column are significantly (*p* < .05) different. Three samples (C_1_, C_2,_ and C_3_) were selected randomly to investigate the variations on coagulant concentration and temperature.

The titratable acidity of the three soft cheese samples was in agreement with the findings of Uaboi‐Egbenni et al. (2010) who have found TA of 0.121 for a cheese made from lactic acid bacteria and *C. procera* extract. Although the result of pH was agreement with the findings of Akinloye and Adewumi (2016), who have found out pH values of 6.47 for cheeses made from *C. procera* extract.

### 3.5 Cheese yield, clotting time, and hardness of cheese

Actual yield (Ya) ranged from 14.13 to 17.89, and highest actual cheese yield was found 18.50 kg cheese/ 100 kg milk for cheese coagulated by commercial rennet. The average coagulating time ranged between 14 and 16 min, and hardness of soft cheese varied from 8.21N to 14.93N where the control sample shows 7.4 N (Table 7).

**Table 7:** Clotting time, cheese yield, whey volume, and hardness of soft cheese

Samples	Yield of cheese (g)	Actual cheese yield (kg cheese /100 kg milk)	Whey volume (ml)	Clotting time (min)	Hardness of cheese (*N*)
C_1_	156.515 ± 4.25^d^	15.42 ± 0.82^d^	809.00 ± 4.3^c^	16.36 ± 0.9^a^	8.21 ± 1.52^e^
C_2_	151.22 ± 4.04^d^	14.90 ± 0.98^e^	858.40 ± 6.4^a^	15.58 ± 1.05^b^	14.93 ± 4.37^a^
C_3_	172.53 ± 3.20^b^	17.00 ± 0.66^b^	742.80 ± 3.2^e^	15.21±0.26^b^	10.45 ± 1.07^c^
C_4_	143.46 ± 4.73^e^	14.13 ± 0.43^e^	851.50 ± 4.31^a^	16.00 ± 0.84^a^	9.70 ± 1.43^d^
C_5_	162.73 ± 2.98^c^	16.03 ± 0.53^c^	775.80 ± 4.23^d^	15.32 ± 0.99^b^	10.45 ± 1.98^c^
C_6_	170.98 ± 2.00^b^	16.85 ± 0.76^c^	710.10 ± 5.24^g^	15.08 ± 0.98^b^	10.45 ± 1.05^c^
C_7_	145.66 ± 4.44^e^	14.35 ± 0.48^e^	820.80 ± 4.96^b^	15.53 ± 1.09^b^	13.81 ± 2.01^b^
C_8_	177.80 ± 2.06^b^	17.52 ± 1.03^b^	720.60 ± 6.02^f^	14.49 ± 1.3^c^	10.45 ± 1.05^c^
C_9_	181.59 ± 1.87^a^	17.891 ± 1.10^b^	715.20 ± 4.22^f^	14.30 ± 1.2^c^	9.33 ± 2.00^d^
C_10_	187.76 ± 1.95^a^	18.50 ± 1.43^a^	601.90 ± 5.69^h^	ND	7.40 ± 1.56^f^

Note

Abbreviation: ND, not determined.

All results are means ± *SD*. Values with the same subscripts of similar column are significantly (*p* < .05) different, where C_10_ (Cheese 10) represents control sample produced using commercial rennet.

Actual cheese yield (kg cheese /100 kg milk) result of current study was somehow in agreement with the findings of Benyahia Krid et al. (2016), and it was revealed 17.69 g/100 g yield of cheese. Despite the fact that, findings of Chikpah and Teye (2015) reported that yield of soft cheeses was 203.1, 214.3, and 237.4 g/L. These yields reported by Chikpah and Teye (2015) were for soft cheeses produced at concentrations of 2, 5, and 7 g, respectively. Results of cheese yield vary compared to other researchers findings, whereas the current study soft cheese yield was expressed in actual yield which includes amount of starter culture used for manufacturing.

The clotting time for *C. procera* dried leaves with 2–7 g dose was ranged from 27 to 29 min (Chikpah and Teye (2015). The shortest clotting time was observed while used starter culture for cheese production. The clotting time differed significantly (*p* < .05) among different temperature (30, 45, 60°C) and different concentration (2, 6, 10 g) treatments. Generally, the coagulating time reduced with increasing concentration of *C. procera* extract as well as extraction temperature.

The texture of dairy foods is as important as flavor and color. The dairy manufacturers are now using texture as a key differentiating quality to improve a product's overall consumer appeal. The texture profile of soft cheese prepared from *C. procera* coagulant has harder texture profile compared with control sample. The values of hardness of soft cheese prepared from *C. procera* coagulant obtained in this study were higher compared with the findings of García et al. (2012), who reported hardness of 9.51N and 7.99N for a cheese made from the plant extracts of *Cynara Cardunculus* subspecies *Cardunculus* and *Cynara cardunculus* sub species *Flavescens*, respectively. The difference was being by the type of plant origin, differences in environmental conditions, and biochemical composition of extract.

### 3.6 Microbiological quality of fresh cheese

The three cheese samples (1, 2, and 3) show reduced amount of microbial load comparing to sample 4 (Table 8). Results of TPC control sample (sample 4) were greater than sample 3, sample 2, and sample 1, respectively. Yeast and mold counts of cheese samples produced by *C. procera* coagulant and commercial calf rennet were superior than sample 3, sample 2, and sample 1, correspondingly (Table 8). This could be because of the sanitation adopted during milking, transportation, storage, and processing of cheese production. Even though the results for *E. coli* for all samples were detected below 1 * 10^1^.

**Table 8:** Microbiological quality characteristics of soft cheese samples

Parameter	Sample 1 (Cfu/g)	Sample 2 (Cfu/g)	Sample 3 (Cfu/g)	Sample 4 (Cfu/g)
TPC (total plate count)	1*10^3^	2*10^3^	4*10^3^	10*10^5^
Yeast and mold	2*10^3^	2*10^3^	3*10^3^	1*10^4^
*E. coli*	<1*10^1^	<1*10^1^	<1*10^1^	<1*10^1^

Note

All values are means of triplicate, where sample 1 (C_1_) was cheese produced at low dose and extraction temperature (2g at 30°C), sample 2 (C_5_) was cheese produced at medium dose and extraction temperature (6g at 45°C), sample 3 (C_9_) was cheese produced at high dose and high extraction temperature (10g at 60°C), sample 4 is a control cheese sample processed by commercial rennet.

C_1_, C_2,_ and C_5_ are selected cheeses made at lowest, medium, and highest extraction temperature and various dose of crude calotropin extract.

In this count, <1 × 10^1^ is the standard reporting format for plates from all dilution of the sample which has no colonies.

Yeast and mold counts of the three cheese samples were in conformance with the findings of Seifu, (2013) who reported yeast and mold counts of metata ayib are ranging from 1.2 × 10^2^ to 6.1 × 10^3^ cfu/g, whereas TPC of metata ayib are ranging from 2.5 × 10^5^ to 6.9 × 10^7^cfu/g and 6.35 log10 cfu/g which is much higher than reported by the current study. In the current research findings, the TPC values of soft cheese samples were lees than that of the international standard set by Codex Alimentarius (1968).

Microbial load of cheese samples (1, 2, and 3) were significantly (*p* < .05) affected by *C. procera* crude extract. Furthermore, samples 1, 2, and 3 revealed reduced TPC comparing to sample 4 which was a control cheese sample processed by commercial rennet. This is because the antimicrobial property of plant extract inhibits the microbial load of the cheese. In a nutshell, the results of microbiological quality characteristics of soft cheese samples obtained in this study were in agreement with the standard set by codex alimentarius commission.

### 3.7 Sensory quality evaluation of soft cheeses

**Figure 3:**
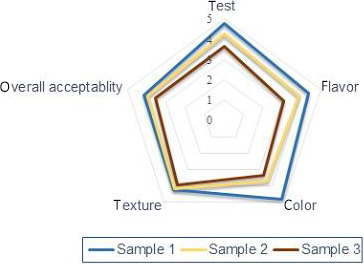
Radar plot of sensory quality evaluation of soft cheese. Where sample 1 (C1) was cheese produced at low dose and extraction temperature (2 g at 30°C), sample 2 (C5) was cheese produced at medium dose and extraction temperature (6 g at 45°C), sample 3 (C9) was cheese produced at high dose and high extraction temperature (10 g at 60°C)

The sensory evaluation of the cheese produced from the different concentration of crude enzyme extract of *C. procera* is shown in Figure 3. Taste, flavor, color, texture, and overall acceptability of all the cheese products were significantly (*p* < .05) affected by the coagulants, whereas texture of cheese samples was not significantly different. The cheese sample produced at low dose (2 g) and extraction temperature (30°C) revealed better sensory quality acceptability. Generally, overall acceptability of the products reduced when the coagulant concentration amount increased. Sample 1 had better taste, flavor, texture, and color, as well as high overall acceptability. A good milk‐clotting enzyme is characterized by a high specific caseinolytic activity and a low general proteolytic activity, since the proteolysis strongly affects the sensory properties of cheese.

## 4 CONCLUSIONS

The research focuses on the use of the enzyme *calotropin* extract, recovered from *C. procera* plant, as milk coagulant alternative to rennet for the production of fresh cheese. The research findings indicated that *Calotropis procera* extract can be used as an alternative coagulant instead of using animal rennet for fresh cheese manufacture in the East African context of pastoral and agro‐pastoral areas where surplus milk and the *C. procera* plant are available. Converting the whole milk into soft cheese can preserve the milk and reduces postharvest losses of milk in unreached areas to collect milk. Based on *calotropin* potential milk‐clotting activity and toxicity result, *C. procera* extract provides valued milk coagulation for soft cheese manufacture. This research study also confirms that the soft cheese produced using *calotropin* enzyme as milk coagulant revealed no adverse effects of toxicity tested on albino rates to the highest dose. Moreover, cheese produced by *C. procera* coagulant provided bounteous amount of nutrients which can be a good source of healthy food to improve food and nutrition insecurity. In conclusion, *calotropin* enzyme used for soft cheese production was safe from toxicity point of view, and the extracted enzyme potentially uses for other enzyme‐based nutraceutical and functional food products manufacturing via innovative industrial biotechnology. This information can provide opening the possibility for the production of soft cheese varieties with additional plant flavors.

This research outcome will provide development of plant rennet for commercial use as a cheap source of bovine rennet substitute in the dairy industry and could be used in future for gene encoding. This research information can enhance the cultivation of *C. procera* plant commercially for the production of a soft cheese as cheap and available milk‐clotting enzyme. Further research on milk‐clotting properties of plant rennet including *calotropis procera* and their enzymatic, rheological, and sensory role in cheesemaking, and evaluation of plant crude enzyme extracts for application in food processing industries besides medicinal potential of bioactive substances are essential.

## 5 STUDIES IN HUMANS AND ANIMALS

This research does not involve the use of human subjects performed by the any of the authors. All mice experiments comply with the guidelines and carried out in accordance with the U.K animals (Scientific Procedures) Act, 1986, and associated guidelines for the care and use of Laboratory animals (NIH Publications No. 8023, revised 1978). The authors indicate that informed consent was obtained for experimentation with animals. Overall, for toxicity test of the extract and manufactured cheese products a total of thirty‐five healthy female mice (weighing 20‐25g) were obtained from animal house of Ethiopian Public Health Institute. They were kept under standard conditions (at a temperature of 21 ± 2℃, with 12 hours’ light/12 hours’ dark cycle) and provided with free access to standard pellet laboratory diets and drink tap water unlimitedly.

## 6 COMPLIANCE WITH ETHICAL STANDARDS

The study conforms to the declaration guideline reviewed and approved by the research ethics committee of the Addis Ababa Institute of Technology. Informed consent was waived by the board, and the research is in line with the international recommendations for the ethical standards.

## CONFLICT OF INTEREST

The authors declare no conflict of interest. The author affirms that this manuscript is an honest, accurate, and transparent account of the study being reported.
